# Stroboscopic visual training: The potential for clinical application in neurological populations

**DOI:** 10.1371/journal.pdig.0000335

**Published:** 2023-08-23

**Authors:** Julia Das, Richard Walker, Gill Barry, Rodrigo Vitório, Samuel Stuart, Rosie Morris

**Affiliations:** 1 Department of Sport, Exercise & Rehabilitation, Northumbria University, Newcastle upon Tyne, United Kingdom; 2 Northumbria Healthcare NHS Foundation Trust, North Tyneside General Hospital, North Shields, United Kingdom; University of Cagliari: Universita degli Studi Di Cagliari, ITALY

## Abstract

Visual problems are common in people who have neurological injury or disease, with deficits linked to postural control and gait impairment. Vision therapy could be a useful intervention for visual impairment in various neurological conditions such as stroke, head injury, or Parkinson’s disease. Stroboscopic visual training (SVT) has been shown to improve aspects of visuomotor and cognitive performance in healthy populations, but approaches vary with respect to testing protocols, populations, and outcomes. The purpose of this structured review was to examine the use of strobe glasses as a training intervention to inform the development of robust protocols for use in clinical practice. Within this review, any studies using strobe glasses as a training intervention with visual or motor performance–related outcomes was considered. PubMed, Scopus, and ProQuest databases were searched in January 2023. Two independent reviewers (JD and RM) screened articles that used strobe glasses as a training tool. A total of 33 full text articles were screened, and 15 met inclusion/exclusion criteria. Reported outcomes of SVT included improvements in short–term memory, attention, and visual response times, with emerging evidence for training effects translating to balance and physical performance. However, the lack of standardisation across studies for SVT protocols, variation in intervention settings, duration and outcomes, and the limited evidence within clinical populations demonstrates that further work is required to determine optimal strobe dosage and delivery. This review highlights the potential benefits, and existing research gaps regarding the use of SVT in clinical practice, with recommendations for clinicians considering adopting this technology as part of future studies in this emerging field.

## Author summary

Visual problems are common in people who have neurological disease and can affect balance and walking. Vision therapy could be a useful intervention for visual problems in various neurological conditions such as stroke, head injury, or Parkinson’s disease. Stroboscopic visual training (SVT) is a type of visual training that involves the use of specialist glasses equipped with liquid crystal technology lenses. These lenses regulate visual information by producing an intermittent clear and opaque flicker effect without light or flash being used. Training with these glasses has been shown to improve aspects of visuomotor and cognitive (thinking) performance in healthy populations, but approaches to training vary. Here, we examine the use of strobe glasses as a training intervention with the aim to help develop protocols for use in clinical practice. We found evidence that SVT can improve visual skills, with the potential to enhance aspects of physical performance such as balance. However, we also found a lack of standardisation across studies for SVT protocols, and there was limited evidence for use of SVT within clinical populations. Our review not only highlights the potential benefits of using SVT in clinical practice but also emphasises where there are gaps in the current research. Based on our finding, we have made recommendations to help guide future research in this emerging field.

## 1. Introduction

The concept of visual training (also known as vision therapy) as a noninvasive intervention for visual impairment has been around for almost a century. Unlike lenses, prisms, filters, and occluders, which compensate for vision problems, or eye surgery that alters the anatomy of the eye or surrounding muscles, visual training uses sensory-motor-perceptual stimulation to enhance visual skills (such as eye movement control and eye coordination) as well as visual processing [1–4]. Vision training has also been used to enhance visual performance in healthy individuals [5,6], where oculomotor exercises or interruption of normal visual input have been associated with changes in motor function and improved physical performance [7–9]. Stroboscopic visual training (SVT) is a form of vision training aimed at improving aspects of visual and cognitive-perceptual performance [1]. SVT has taken a number of different forms since its first use over 20 years ago from the application of strobe lights in dark settings to digitally controlled eyewear [7,10]. Research has indicated that SVT may result in improved visual–motor actions such as driving performance [11], as well as reductions in symptoms that result from visual–motor conflict, such as motion sickness [12,13].

Interest in the use of SVT as a sports training tool began a decade ago following the findings of a study by Appelbaum and colleagues, which showed improved attention and motion detection following SVT in cohorts of university team–based athletes [9]. Subsequent studies using SVT in healthy individuals have shown promising results in improving visual acuity [14], visual attention [15], and visuomotor performance [16–18]. While research has yet to identify the exact mechanisms of action driving SVT, the approach is based on the basic premise that by intermittently disrupting vision, individuals reduce their reliance on visual feedback. This may lead to increased sensitisation and better visual skills when they return to normal visual conditions, as well as resulting in more effective utilisation of other sensory modalities, such as kinaesthetic awareness and auditory information [1]. Indeed, stroboscopic visual perturbation has been shown to be a valid assessment method for sensory dependence, promoting sensory reweighting and improving balance control [19–21].

Visual problems are common in people who have neurological injury or disease, such as stroke and traumatic brain injury or Parkinson’s disease [22], with deficits linked to postural control and locomotion impairment [23]. Vision therapy, particularly SVT, could be a useful intervention for visual impairment in various neurological conditions. Indeed, vision therapy has been used for treatment of binocular and accommodative dysfunctions, strabismus, amblyopia, oculomotor dysfunctions in acquired brain injury and mild traumatic brain injury (concussion) and visual information processing disorders [4,24]. However, it is unclear how to implement visual training to intervene in visual impairment within clinical populations. For example, within mild traumatic brain injury, visual training duration varies depending on the diagnosis and the patient, typically ranging from several months to longer periods of time [4]. Clinicians or researchers who wish to use or investigate visual training methods are left without any clear guidance on how to implement such methods. Therefore, a structured review with recommendations on specific visual training methods is warranted. A previous review of the impact of SVT showed initial efficacy of SVT in improving visual performance in healthy individuals [1], but little information about the SVT methodology was reviewed. In order to develop robust interventional protocols, it is important to understand how SVT has been effectively deployed, such as what cohorts it has been used in (healthy, clinical, etc.), duration of training, strobe frequency, type of SVT device, exercises coupled with SVT, etc.

The purpose of this review is to identify all previously peer-reviewed research that has investigated the use of strobe glasses as a training intervention with a visual or motor performance related outcome in healthy and clinical populations. The review aimed to (1) examine the methods involved in SVT; (2) present the commonly reported outcomes and explore how SVT influences these; and (3) provide clinical and future research recommendations.

## 2. Methods

### 2.1 Search strategy

**Fig 1:**
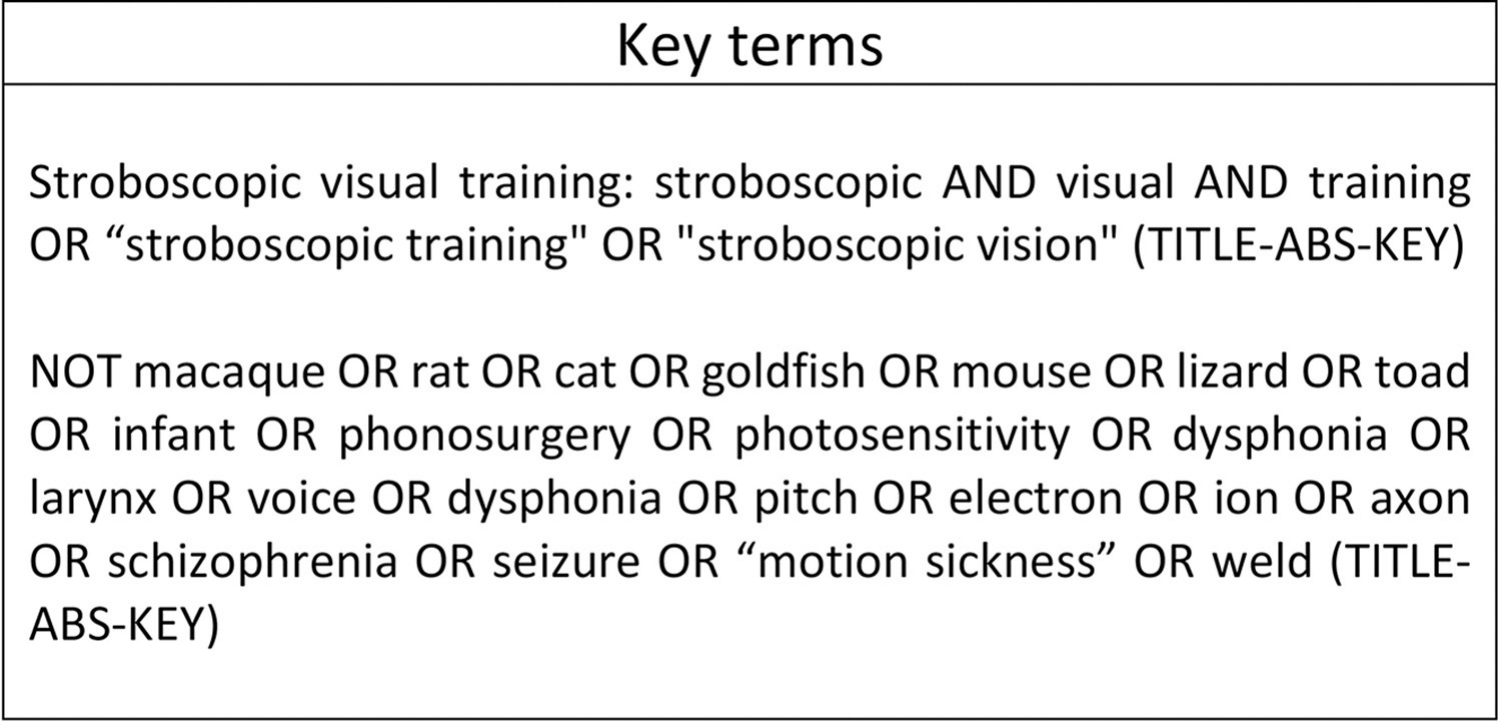
Search strategy used to screen for relevant articles included in this review. This illustrates the key terms used for this review.

An initial online search of 3 databases (PubMed [PM], Scopus [S], and ProQuest [PQ]) was carried out in January 2023 using the search terms shown in Fig 1. The search was limited to full journal articles written in the English language published from 1950 to January 2023. Duplicates were deleted and an initial title screen was performed by the reviewer (JD). After the initial title screen, abstracts were uploaded to the Rayyan application (https://www.rayyan.ai/) and reviewed by 2 independent reviewers (JD and RM). Full text screens were carried out if it was not clear from the title or abstract whether the study met the review criteria.

### 2.2 Inclusion and exclusion criteria

Articles were included if they reported use of stroboscopic vision (SV) as a training intervention. Only original research articles using human participants were included, utilising stroboscopic eyewear during a defined training period to produce intermittent vision and impact on visual and/or motor performance. Studies were excluded if they investigated the immediate influence of SV on task performance [19–21,25–31], if SVT was applied as part of a multifaceted visual training intervention [32–34], if the study used alternative methods of delivering strobe effects (e.g., vertical cyclinders) [35], or if it investigated effects on motion sickness without a visual or functional motor outcome [12]. Three studies were excluded on the basis that they were theses generated from graduate projects and not fully peer reviewed [14,36,37]. A further 2 studies were excluded on the basis that they were exploring changes in cortical activation as a result of SVT [38,39]. Any abstracts, commentaries, discussion papers or review articles, or conference proceedings were also excluded.

### 2.3 Data extraction

Data were extracted and synthesised into table format by the reviewer (JD), and a second reviewer (RM) confirmed the entered data (Tables 1–4). Information included participant groups and characteristics, type of eyewear used (including strobe settings where available), study protocols, and key findings.

**Table 1:** Participant characteristics, diagnosis, eligibility criteria and design, and aims of the reviewed studies.

Author	Participants	Diagnosis	Inclusion Criteria	Exclusion Criteria	Study Design and Aims
**Appelbaum et al. (2011)**	157 participants University students and athletes	None: healthy population	Not recorded	H/O Seizures Migraines Sensitivity to light	• Pretest posttest study • Effects of SVT on: 1. Motion sensitivity 2. Peripheral vision and dual-task attention 3. Sustained attention
**Appelbaum et al. (2012)**	*Experiment 1*: 77 university students SVT group (*n* = 40) Control group (*n =* 37) *Experiment 2*: 33 university students	None: healthy population	Not recorded	Not recorded	• Pretest posttest study 1. Immediate effect of SVT on visual memory 2. 24-hour retained effect of SVT on visual memory
Bennett et al. (2018)–Experiment 2*	52 adults - mean age 22.3 years 3 experimental groups: Normal vision Vapor Strobe PLATO 1 control group (no practice)	None: healthy population	Normal/corrected to normal vision	>7,500 hours playing computer games	• Experiment 2 of a pretest posttest study • Effect of SVT on acquisition of multiple object avoidance task
**Braly and DeLucia (2020)**	*Experiment 1*: 60 university students (30 M, 30 F) - age range: 18–41 years *Experiment 2*: 60 university students (30 M, 30 F) - age range: 18–35 years	None: healthy population	• Normal/corrected to normal vision	Not recorded	• Pretest posttest study • Effects of SVT on: 1. Time-to-collision judgements of approaching objects 2. Time-to-collision judgements of laterally moving objects
**Ellison et al. (2020)**	62 male mixed ability sports participants - age 19–25 years SVT group (*n =* 31) Control group (*n =* 31)	None: healthy population	Novice to the eye–hand coordination task	Epilepsy	• Pretest posttest study • Effects of SVT on eye–hand coordination
**Hülsdünker et al. (2019)**	10 top level badminton players (1 M, 9 F) - mean age: 23 years SVT group (*n* = 5) Control group (*n* = 5)	None: healthy population	Regular participation in national and international badminton tournaments Visual acuity: = 20/20+ (on Landolt test)	H/O migraine Epileptic seizures Any neurological disorder (not specified) that would contraindicate SVT	• Longitudinal pretest posttest study • Effects of SVT on visuomotor performance and neural visual function
**Hülsdünker et al. (2021): Part 1 and Part 2**	32 young elite badminton athletes (21 M, 11 F) - Mean age: 13.7 years SVT group (*n* = 16) Control group (*n =* 16)	None: healthy population	Competitive at national-level badminton	History of epilepsy, migraine History of neurological or psychiatric disorder	• Longitudinal pretest posttest study • Part 1: Short- and long-term effects of SVT on reaction speed in elite athletes • Part 2: Effect of SVT on reaction time
**Kim et al. (2021)**	73 adults (42 M, 31 F) - Mean age: 28.9 years Balance training group (*n* = 26) SVT group (*n =* 26) Control (*n* = 26)	CAI	International Ankle Consortium inclusion criteria recommendations Mental and physical autonomy	History of other MSK in lower limbs Self-reported vestibular or balance dysfunction Acute ankle sprain within last 6 weeks Recent surgery Epilepsy or history of seizures	• Pre-test post- test single-blind randomised controlled study • Effects of SVT on dynamic balance
**Mitroff et al. (2013)**	11 male ice hockey players - age range: 20–27 years SVT group (*n* = 6) Control group (*n =* 5)	None: healthy population	Professional ice hockey players	Not recorded	• Pilot pretest posttest study • Effects of SVT on ice hockey puck placement
**Shalmoni and Kalron (2020)**	26 PwMS (10 M, 16 F) - mean age: 47.9 years	Multiple Sclerosis (MS)	Diagnosed with MS Age: 25–55 years EDSS^+^: 2.0–5.5 Ability to understand and execute simple instructions	H/O migraine Epileptic seizures Contraindication to physical activity Cognitive impairment Pregnancy Severe uncorrected visual deficits MS relapse /corticosteroid/ disease modifying treatment within 90 days Depression Participating in other cognitive impairment trial	• Pretest posttest single-blind randomised cross-over study • Effects of SVT on cognitive function, gait, and static balance performance in PwMS.
**Smith and Mitroff (2012)**	30 university participants (12 M, 18 F) SVT group (*n =* 15) Control group (*n =* 15)	None: healthy population	First come first served	Not recorded	• Pretest posttest study • Effects of SVT on anticipatory timing
**Symeonidou and Ferris (2022)**	40 adults (20 M, 20 F) - Mean age: not recorded SVT group (*n* = 20) Control group (*n* = 20)	None: healthy young adults	None specified	Neurological, orthopaedic, or musculoskeletal condition Lower limb surgery	• Longitudinal pretest posttest randomised parallel group trial • Effects of SVT on balance
**Wilkins and Gray (2015)**	30 undergraduate athletes (18 M, 12 F) - mean age: 22.5 years	None: healthy population	Normal/corrected to normal vision Member of a sports club/team Between 5–20 years sport experience	No exclusion criteria	• Pretest posttest study • Effects of SVT on visual attention, motion perception, and catching performance
**Wilkins et al. (2018)**	3 male elite youth football keepers 3 matched controls - age range: 16–19 years	None: healthy population	Elite level In full-time football training	Not recorded	• Pilot mixed methods case study • Effects of SVT on visual and perceptual skill • Qualitative exploration of participant experience of SVT
**Zavlin et al. (2019)**	22 adult medical students (14 M, 8 F) - mean ages 23.6–24.2 years SVT group (*n* = 11) Control group (*n =* 11)	None: healthy population	Enrolled medical students No prior surgical experience	Not recorded	• Pretest posttest study prospective randomised-control trial • Effects of SVT on proficiency of surgical task

*Study reported 2 experiments. Experiment 1 examined effects of SV on performance of a multiple object tracking task and did not meet the eligibility criteria for this review; therefore, only findings from Experiment 2 will be considered.

^+^EDSS, Expanded Disability Status Scale.

**Table 2:** Stroboscopic eyewear, settings, training protocol, test points, and key findings.

	Strobe settings	Frequency/duration of training	Total strobe time (min)	Intervention/task	Key findings
**Nike Vapor Strobes**					
**Appelbaum et al. (2011)**	Modulated frequency Levels 1–6 (6–1.75 Hz) Modulated frequency Levels 2–4 (5–3 Hz)	*Cohort 1* (In lab) 2 or 4 sessions × 27 minutes *Cohort 2* (Ultimate frisbee) 4 sessions × 20–28 minutes *Cohort 3* (Varsity football) 9 or 10 sessions × 15–30 minutes	54–300	Catching drills Throwing drills in stationary and running situations. Speed and agility drills	Improved motion detection* Improved central attention* No change in sustained attention
**Appelbaum et al. (2012)**	*Experiment 1*: Fixed frequency or modulated frequency depending on drill Levels 1–6 (6–1.75 Hz) Experiment 2 Fixed frequency or modulated frequency depending on drill Levels 1–6 (6–1.75 Hz)	*Cohort 1* (In lab) 2 sessions × 27 minutes *Cohort 2* (Varsity soccer) 6 or 7 sessions × 15–45 minutes *Cohort 3* (Varsity basketball) 5 or 6 sessions × 15–40 minutes (In lab) 2 sessions × 27 minutes	54–315 54	Turn and catching drills Passing and dribbling drills Agility and ball handling drills Turn and catching drills	Improved short-term memory capacity* Retention of increased short-term memory capacity*
**Bennett et.al. (2018)**	Fixed frequency Level 6 (1.8 Hz)	Single session Time not recorded	Approximately 30 minutes	Computer-based multiple object avoidance task	Improved acquisition of multiple object avoidance task*
**Ellison et al (2020)**	Fixed frequency Level 3 (4 Hz)	Single session × 5–7 minutes	5–7	Electronic light board	Improved EHC at all 3 retention test points*
**Mitroff et al. (2013)**	Modulated frequency Levels 1–8 (1–6 Hz)	16 sessions × ≥10 minutes	160	Ice hockey skills on/off ice, e.g., skating, passing, balance, conditioning	Improved passing/shot accuracy*
**Smith and Mitroff (2012)**	Fixed frequency Level 3 (4 Hz)	Single session × 5–7 minutes	5–7	Anticipation practice using Bassin Timer—(participants press button on wired response wand)	Improved anticipatory timing accuracy immediately after training* Improved consistency in timing estimates immediately after training and 10 minutes later*
**Wilkins et al (2018)**	Modulated frequency Levels 1–8 (1–6 Hz)	7 weeks 14 sessions x 45 minutes; 1 x 5 minutes	635	Tennis ball catching drills Goalkeeper specific drills using football	Improved visual response time~ SVT had no effect on attention, anticipation, and hand–eye coordination
**Senaptec strobes**					
**Hülsdünker et al. (2021) Parts 1 and 2**	Preprogrammed frequency 15–8 Hz (duty ratio: 50%–70%)	10 weeks 10 sessions × 10–15 minutes	11.1–85.6	Court-based badminton drills Exercises with ball-machine	Improved visuomotor reaction time*
**Kim et al. (2021)**	Modulated frequency Levels 1–8 (1–6 Hz)	6 weeks 18 sessions × 20 minutes	360	Circuit of 6 progressively more difficult balance exercises	Improved anterior reach* Improved perceived ankle stability*
**Symeonido and Ferris (2022)**	Fixed frequency of opaque phase 0.1 Hz	Single session × 30 minutes	30	Walking on a treadmill mounted balance beam	Improved dynamic balance (as indicated by a reduction in step-offs)*
**Zavlin et al. (2019)**	Fixed frequency Level 4 120 Hz	5 weeks 5 sessions Time not recorded	Not known	Surgical tasks (knot ties, sutures, stitches)	Improved surgical performance*
**MJ Impulse Strobes**					
**Hülsdünker et al. (2019)**	Modulated frequency 5–6 Hz (duty cycle 50%–70%)	12–20 × 12–15 minutes	108–300	Badminton-specific training drills	Improved visuomotor performance* No effect on neural function.
**Visionup glasses**					
**Shalmoni and Kalron (2020)**	Fixed frequency 30 Hz Duty ratio: 50%	2 sessions Separated by 2-week washout period 1 × 40–50 minutes with strobes 1 × 40–50 minutes normal vision	40–50	Ball catching drills	Improved information processing speed* No differences in gait and balance outcomes
**PLATO goggles**					
**Braly (2020)**	Fixed frequency Level 3 (4 Hz)	Single session 5–7 minutes	5–7	Screen-based task involving moving objects	No significant effect on time-to-collision judgements
**Wilkins and Gray (2015)**	Modulated frequency 8–1 Hz	8 × 20 minutes 1 × 5 minutes	165	Ball catching drills	Improved motion-in-depth sensitivity No differences found between fixed strobe and variable strobe settings

* indicates a statistically significant improvement compared to control group

~No statistical analysis performed.

**Table 3:** Posttraining test points.

Study	Test points						
	Immediate	10 minutes	24 hours	10 days	2 weeks	4 weeks	6 weeks
**Appelbaum et al. (2011)**	**✓**						
**Appelbaum et al. (2012)**	**✓**		**✓**				
**Braly et al. (2020)**	**✓**						
**Bennet et al. (2018)**	**✓**						
**Ellison et al. (2020)**	**✓**	**✓**		**✓**			
**Hülsdünker et al. (2019)**	**✓**						
**Hülsdünker et. al. (2021)**	**✓**						**✓**
**Kim et al. (2021)**	**✓**						
**Mitroff et al. (2013)**			**✓**				
**Shalmoni and Kalron (2020)**	**✓**						
**Smith and Mitroff (2012)**	**✓**	**✓**		**✓**			
**Symeonido and Ferris (2022)**	**✓**				**✓**		
**Wilkins and Gray (2015)**	**✓**						
**Wilkins et al. (2018)**	**✓**					**✓**	
**Zavlin et al. (2019)**	**✓**						

**Table 4:** List of measured variables in the 15 studies.

	Visual measures	Motor performance measures
**Symeonido and Ferris (2022)**		Dynamic balance
**Kim et al. (2021)**		Dynamic balance
**Hülsdünker et al. (2021) Part 1**	Visual perception and reaction	Sport-specific performance task (ball-racquet)
**Shalmoni and Kalron (2020)**	Cognitive function Verbal/nonverbal memory Executive function Visual spatial processing Verbal function Attention Information processing speed Motor skills	Gait and balance Walking speed Stride time Stride length Cadence Static balance
**Ellison et al. (2020)**	Reaction time Hand–eye coordination Visual search	
**Braly et al. (2020)**	Time-to-collision judgements (of approaching and laterally approaching of objects)	
**Zavlin et al (2019)**		Surgical task performance (suturing)
**Hülsdünker et al. (2019)**	Visual processing	Sport-specific performance task (badminton smash defence)
**Bennett et al. (2018)**	Visual attention	
**Wilkins et al. (2018)**	Processing speed Divided attention Selective attention Sustained attention Anticipation Visual response speed Hand–eye coordination Response inhibition Visual spatial working memory	
**Wilkins et al. (2015)**	Motion-in-depth sensitivity Processing speed Divided attention	Tennis ball catching
**Mitroff et al. (2013)**		Sport-specific performance task (ice hockey passing/shot accuracy)
**Smith et al. (2012)**	Eye–hand coordination Anticipation	
**Appelbaum et al. (2012)**	Short-term visual memory	
**Appelbaum et al. (2011)**	Motion sensitivity Divided attention Multiple object tracking	

## 3. Results

### 3.1 The evidence base

**Fig 2:**
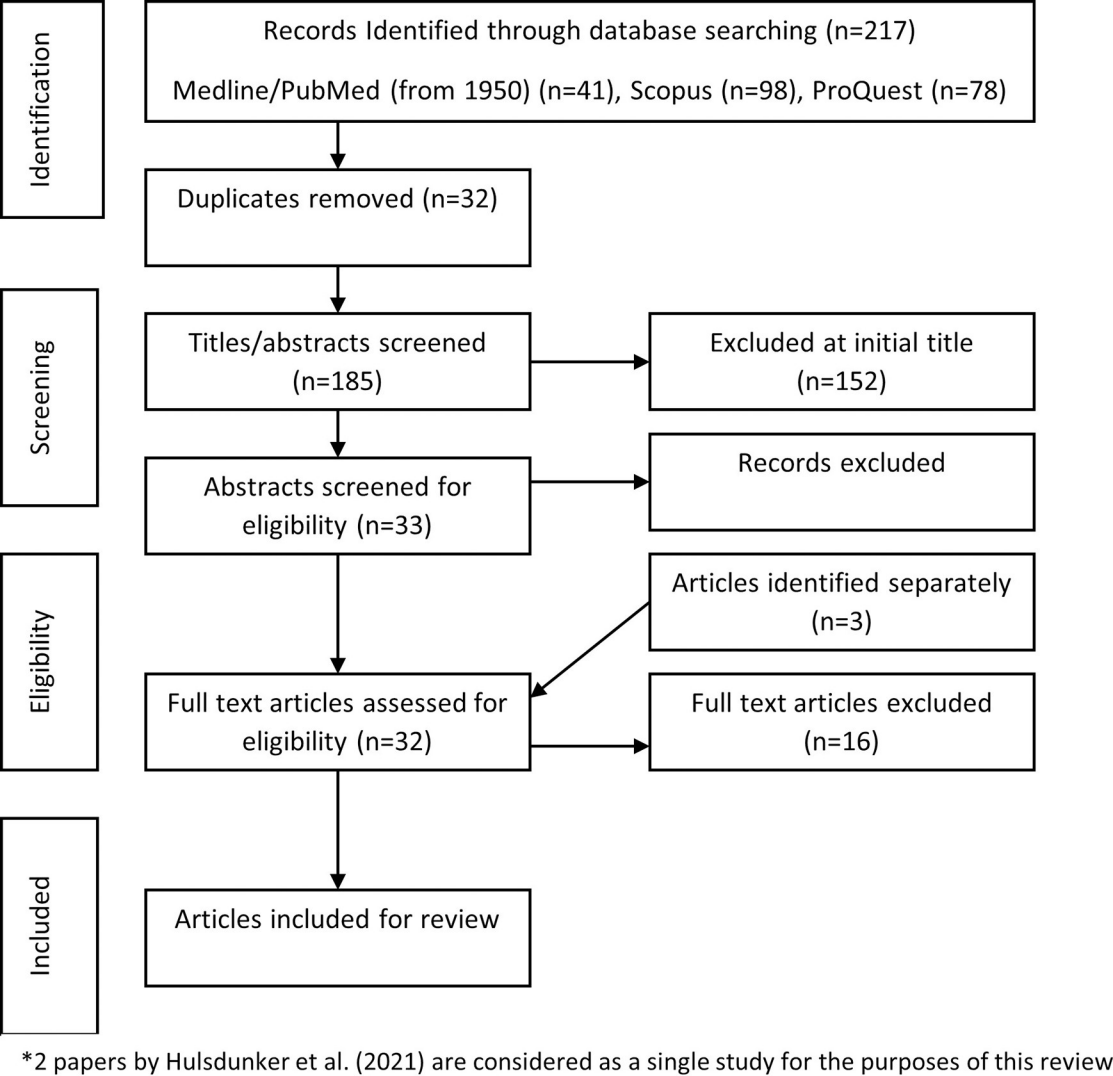
PRISMA Search Strategy (Search updated January 2023).

The search strategy generated a total of 217 papers (Fig 2). After duplicates, a total of 185 papers were yielded from the search. An initial title/abstract screening by the first reviewer (JD) resulted in 33 articles of interest plus an additional 3 papers identified from separate sources. Four papers were excluded following the abstract screen leaving 32 papers requiring full text review by the first and second reviewers (JD and RM). A consensus was made for inclusion of 16 articles for review by the study team. Two papers by Hülsdünker and colleagues reported separately on the same sample of 32 elite badminton athletes and are therefore considered as a single study for the purposes of this review [40,41].

### 3.2 Study populations

#### 3.2.1 Participants

Recruitment was predominantly through university student cohorts or professional/elite sports teams, with participants aged in their late teens or twenties; 2 of these studies involved participants with a history of ankle injury, but participants were otherwise fit and healthy. One study explored the immediate effect of SVT on people with a neurological condition (multiple sclerosis; MS) [42]. Both male and female were recruited to the majority of studies (*n =* 12) except for 3 studies that recruited only male participants [18,43,44]. Three papers did not report specific gender characteristics [9,15,45]. (Table 1).

#### 3.2.2 Eligibility criteria

Seven studies recorded no exclusion criteria for their participants [15,43,44,46–49]. The remaining 8 studies identified seizures/epilepsy, migraines, and/or a history of neurological disorder as reasons for individuals not to take part (Table 1). Inclusion criteria were generally focused on level of sporting ability except in the case of the later studies where participants were recruited based on an underlying health condition [42] or a musculoskeletal injury [50].

### 3.3 Cohort structure

#### 3.3.1 Sample size

Participant sample sizes varied greatly between the studies with numbers ranging from a pilot study of 3 [44] to the earliest study by Appelbaum and colleagues who had 157 participants [9].

#### 3.3.2 Control groups

All 15 studies included a control group. In an attempt to reduce bias, 4 of these studies had control participants wearing stroboscopic eyewear with the lenses in a permanently transparent (nonstrobing) state [9,15,46] and 1 study used lens-free glasses [42]. Wilkins and Gray applied a constant fast strobe setting to their control group participants in an attempt to combat the potential for differing motivation and effort levels compared to the variable SVT group [48].

### 3.4 Study protocols

#### 3.4.1 Stroboscopic devices

Five different commercially available brands of eyewear device were used to provide stroboscopic visual disruption across the 15 studies (Table 2). The most common devices to feature in the studies were the Nike Vapor Strobe (Nike, Beaverton, OR) (*n =* 7) [9,15,18,43,44,49] and the newer version of the eyewear, the Senaptec Strobes (*n* = 4) [40,41,47,50,51]. Other devices included the Visionup Strobe glasses (*n* = 1) [42], Plato goggles (*n* = 2) [46,48], and MJ Impulse (*n* = 1) [16].

#### 3.4.2 Strobe settings

Of the 15 studies, 7 studies used a single fixed frequency to deliver their stroboscopic training. There were a number of different set frequency settings employed in these studies: 4 Hz [18,46,49], 30 Hz (duty ratio: 50%) [42], 1.8 Hz [45], 0.1 Hz [51], and 120 Hz [47] (Table 2). Seven studies adopted a variable frequency approach [9,16,40,43,44,48,50]. Appelbaum and colleagues used both a fixed and a modulated strobe frequency depending on the drill [15].

#### 3.4.3 Frequency and duration of interventions

Four studies conducted a single, one-off session of SVT lasting from 5 minutes [18,46,49] to a maximum of 50 minutes [42]. The remaining studies (*n =* 11) provided multiple sessions of SVT over periods of up to 10 weeks with Wilkins and colleagues reporting a maximum accumulated total of 635 minutes of strobe time [44] (Table 2). There was also significant variability of training duration within studies. For example, in Appelbaum and colleagues’s study, total strobe time ranged from 54 to 300 minutes [15].

#### 3.4.4 Training intervention

Strobe glasses were worn while undertaking physical tasks such as throwing and catching drills (*n =* 4) [9,15,42,48] or sport-specific activities such as skating and passing drills in ice hockey [43], goalkeeping drills in football [44], or court-based badminton drills [16,40,41]. Two studies applied SVT while participants undertook activities specifically designed to challenge their balance. Kim and colleagues used a multimodal exercise programme performed over 6 weeks, while Symeonidou and colleagues used a single session of balance beam walking [50,51]. Four studies had participants undertaking SVT while performing machine or screen-based reaction time tasks [18,45,46,49], and 1 study had participants practicing surgical tasks under stroboscopic conditions [47] (Table 2).

#### 3.4.5 Posttraining test points

Table 3 demonstrates that across the reviewed studies, 14 posttests occurred immediately after the training with 6 of those studies also recording retention-test data ranging from delays of 10 minutes [18,49], 24 hours [15], 10 days [18,49], 4 weeks [44], and 6 weeks [40]. Mitroff and colleagues conducted a single posttest 24 hours after their last training session [43].

### 3.5 Outcomes

#### 3.5.1 Outcome measures

The majority of studies (*n =* 11) explored SVT outcomes in relation to either a specific visual ability such as reaction timing or hand–eye coordination [18,40,49], visual memory [15], or visual processing [9,16,46,48], or a battery of visual outcomes [42,44] (Table 4). Of the studies that addressed visual ability, some utilised a stand-alone unit such as a Bassin Timer to measure a single visuomotor outcome (anticipatory timing) [49], whereas other studies use computerised software (e.g., MATLAB/Psychophysics Toolbox/NeuroTrax Mindstreams) [9,15,16,42,45], online assessment tools [44,48] or specialist sensory assessment devices [18,40]. Hülsdünker and colleagues used neurophysiologic investigations (via electroencephalography) to identify modulations in the participants’ visuomotor performance and visual perception speed [16].

Of those studies, a further 3 also measured sport-specific performance tasks [16,40,44], and 1 captured gait and balance metrics as a secondary outcome [42]. Four studies included only motor performance measures; dynamic balance [50,51], precision of puck placement in ice hockey [43], and performance of suturing task [47] (Table 4).

#### 3.5.2 Interpretation of outcomes

SVT had a positive effect on several different visual and motor performance outcomes (Table 2), for example, central field motion sensitivity [9], short-term memory capacity [15], processing speed [48], and eye–hand coordination [18]. Conversely, SVT was found to have no significant effect on outcomes that were based on more sustained visual stimuli or stimuli appearing in the peripheral visual field [9].

### 3.6 User experience

Only 2 of the reviewed studies made reference to the participant experience of undertaking SVT [44,48]. Wilkins and colleagues used a custom-made questionnaire that was administered prior to the posttesting session to determine if there were any motivational differences between the fixed strobe and variable strobe training groups [48]. Analysis of these data revealed no significant group differences in enjoyment, motivation, or effort. Wilkins and colleagues conducted semistructured interviews with 3 elite footballers and were the only researchers to include a qualitative component to their data collection [44]. Three themes were identified through thematic analysis of the interview data: (1) the belief that SVT improved visual and perceptual skills (notably reactions, judgement, and focus); (2) the belief that SVT improved on-field goalkeeping performance; and (3) the belief that SVT was both effortful and enjoyable [44]. No studies explored the participant experience of using stroboscopic glasses with regard to comfort and wearability, although Shalmoni and Kalron did make reference to their functionality and ease of use [42].

### 3.7 Safety and adverse events

Reporting of safety issues or adverse events was not included in the aims of any of the studies in this review. None of the studies identified any safety issues or adverse events as a result of participants using strobe glasses.

## 4. Discussion

This structured review examined 15 studies that reported the use of SVT in healthy and clinical populations with the aim to summarise the impact of SVT on visual and/or motor performance. All research studies identified in this review were conducted since 2011, making SVT a relatively new area of study. A major strength of SVT compared to other paper or computer-based visual training tools is that it allows for training to take place in real-world environments. However, its application in a variety of different training contexts has led to significant variability between and within interventions. In this review, we sought to determine what research has been conducted in neurological cohorts, as previous evidence suggests that training visual skills may benefit symptoms in this population.

### 4.1 Participants

With the vast majority of studies including only healthy young participants, the generalisability of findings from this review to older or clinical populations is limited. Recruitment was predominantly through university cohorts or sports teams (with the exception of the study by Shalmoni and Kalron, which was based in an MS specialist medical centre) [42] with sample sizes varying across the studies (i.e., *n =* 6 to 157). A history of epilepsy/seizures, migraines (or more broadly “neurological disorder”) were commonly cited exclusion criteria, although 7 of the reviewed articles made no reference to exclusion criteria, which is perhaps reflective of the fact that the earlier studies were from nonclinical sporting disciplines involving young and healthy individuals. Reporting biases in relation to participant demographics also limit cross-study comparisons. To understand the potential application and efficacy of SVT in clinical populations, further studies conducted in different clinical cohorts and older populations are required. Some authors selected to use nonstrobing eyewear for their control group participants, while others used lens-free glasses or normal vision, but in the absence of a true placebo or blinding option, it is not possible to determine whether motivational effects exist. Indeed, the one study that collected qualitative data regarding SVT revealed that participants believed that their visual, perceptual, and sporting performance all improved as a result of the SVT, indicating the potential of participant bias in favour of SVT [44]. Research is needed to determine whether SVT is perceived as positively by participants from clinical populations in order to identify the most appropriate control methods for future study protocols.

### 4.2 Protocols

#### 4.2.1 Stroboscopic devices

There was a lack of standardisation of stroboscopic instrumentation employed across the studies. The 5 devices used in the studies all had different manufacturer operating levels, so there was no consistent method of reporting the frequency of the strobe effect or “blink rate.” For example, the Senaptec Strobes operate at a level between 1 and 8, with level 1 representing the fastest/easiest setting at a frequency of 6 Hz (calculated according to the number of transparent-opaque cycles per second) and level 8 representing the slowest/hardest setting at a frequency of 1 Hz [52]. In comparison, the Visionup strobes are adjustable by 10 levels whereby level 1 equates to 1 Hz (based on the frequency of the opaque phase), and level 10 equates to 150 Hz [53]. Even for studies utilising the same strobe device, reporting of the strobe setting varied. For example, Kim and colleagues refer to the operating levels of the Senaptec Strobes (i.e., levels 1 to 8) [50], whereas Hülsdünker and colleagues record frequency settings (i.e., 15 Hz to 8 Hz) [40].

The illuminance value (i.e., degree of light transmission provided through the lenses; measured in lux) was only measured by Bennett and colleagues [45], in relation to the Nike Vapor Strobes and the PLATO goggles, but was not reported in any of the other papers in this review… The Visionup and MJ Strobe glasses have the option to adjust the “duty ratio” (or level of brightness of the lenses), to create darker conditions and, therefore, a more challenging visual environment.

Direct comparisons between studies using different brands of eyewear are therefore confounded by the variation in functionality of the different devices, description of lens characteristics, luminance values, and methods of reporting. This was highlighted by Wilkins and Gray who suggested that their use of PLATO goggles (as opposed to the Nike Vapor Strobes) may have contributed to the discrepancy in their findings with previous research [48]. It is evident from this review that more clarity around strobe frequencies and setting levels is required (both from manufacturers of the devices and researchers themselves) to provide clear understanding and accurate interpretation for future clinical application.

#### 4.2.2 Strobe settings

There was a lack of standardisation with regard to (1) the frequency of strobe settings and (2) the use of fixed versus variable strobe frequency settings. Smith and Mitroff examined the effect of SVT delivered at a fixed frequency of 4 Hz (Nike Vapor Strobes: level 3, 100 ms clear: 150 ms opaque) and found that participants in the intervention group had significantly better anticipation immediately after a single session of SVT [49]. Subsequent studies have since used these findings to justify their selection of strobe rate settings [18,46]. The most recent study by Symeonido and Ferris applied a much lower fixed frequency visual perturbation to participants as they walked on a treadmill-mounted balance beam for 30 minutes [51]. Balance performance, in number of step-offs of the beam, improved by 78% for the SVT group on the same day of the training. While the earlier studies in this review support a link between visual stimuli and motor learning, Symeonidou and Ferris attributed their much larger training effect to their use of lower frequency strobe settings [51].

For the 8 studies that adopted a variable frequency approach, training protocols generally started on the easiest (fastest) strobe setting before the strobe rate was reduced in response to the correct execution of training drills or to increase task difficulty over time. The exact duration with which participants trained at each strobe level was poorly documented in the majority of studies, making it difficult to extrapolate the findings (Table 2).

The study by Wilkins and Gray was the only one to directly compare training with a fixed strobe rate and a variable strobe rate [48]. Motion in depth sensitivity significantly improved posttraining, but no significant differences were found between the fixed and the variable rate groups. Further work is therefore required not only to determine whether there is an optimal frequency of strobe setting required for both visual and motor learning to occur but also to establish whether the effects of SVT are greater (or indeed reduced) if participants remain on a fixed strobe frequency [1].

#### 4.2.3 Frequency and duration of interventions

This review demonstrates that there was significant variability regarding the frequency, duration, and number of SVT interventions carried out within the studies (a finding that has been reported across other forms of vision therapy) [54,55]. Interventions ranged from a single session of SVT to multiple sessions over 10 weeks (Table 2). Further complicating interpretation in this review is the lack of clarity provided about the intervention protocols in some papers. For example, Zavlin and colleagues found that SVT had a significant positive effect on the performance of surgical suturing tasks by medical students after 5 sessions of training, but no detail is provided on how long each training session lasted [47]. This makes it difficult to make comparisons between studies because the exact duration of SVT is not known.

In this review, immediate visual improvements were reported after a single session lasting as little as 5 to 7 minutes in some studies [18,49]. This is a significantly lower duration than the 4- to 8-week length of visual training that has been shown to be effective in previous nonstroboscopic research involving athletes [5,56,57]. In contrast with the findings of Smith and Mitroff and Ellison and colleagues [18,49], however, 5 minutes of SVT was not sufficient to improve time to collision judgements of approaching objects in Braly and colleagues’ study [46]. The differing protocols in this review mean that currently no conclusions can be drawn regarding the optimal frequency and duration of SVT that is necessary for a successful outcome in any population.

#### 4.2.4 Training interventions

The lack of standardised training interventions across the studies in this review reflects the fact that SVT allows for training to take place in a variety of “real-world” contexts in addition to more controlled laboratory-based settings. Furthermore, the reporting of the precise details of the training interventions were vague in a number of the studies due to the logistical constraints imposed by using athletic populations, which limits interpretation of their findings [9,15,43]. While some studies using athletic populations did provide a detailed description of the SVT intervention (for example, Hülsdünker and colleagues described 2 badminton-specific protocols), making inferences on these studies remains challenging due to the sport-specific nature of the interventions provided.

Nike developed a series of videos showing exercises for SVT in 2010s. These mostly comprised variations on simple ball catching tasks (such as the wall-ball catch, the front catch, the turn and catch, and the power ball drop). These exercises formed the basis of interventions for 3 of the reviewed studies [42,44,48]. Kim and colleagues also used throwing/catching exercises alongside static and dynamic balance tasks as part of their SVT intervention [50]. Unlike the study by Shalmoni and Kalron who implemented ball drills intended for athletes in an intervention for people with MS [42], to our knowledge, Kim and colleagues’ study is the first to demonstrate how SVT might be used to increase the challenge of standardised exercises, thereby further improving sensorimotor control [50]. Their intervention is supported by the findings of Symeonido and colleagues, who also used SVT to enhance the effects of balance training [51].

Further work is required to determine how best to proceed with the introduction of stroboscopic training protocols in clinical populations. Evidence from this review suggests that one approach would be to use strobe glasses as an adjunct to existing rehabilitation practices whereby interventions are composed of standardised exercise programmes performed with and without stroboscopic glasses.

#### 4.2.5 Posttraining test points

Posttests were carried out within 24 hours in all the studies, providing evidence of the immediate effects of SVT on a range of outcomes. Retention-test data, which are arguably of more interest in relation to the clinical application of SVT, were collected in only 4 of the studies, which limits our understanding of the long-term effectiveness of SVT in any population. Future studies are required to determine whether the effects of SVT last beyond 4 to 6 weeks and, indeed, whether the duration and frequency of SVT affects retention.

### 4.3 Outcomes

Various outcomes were used to assess the efficacy of SVT across the studies, including measures of visual skills (e.g., reaction time, visual memory, processing speed) and motor performance (e.g., balance, sport-specific skills). The paper by Shalmoni and Kalron was one of only 4 studies to include measures of both visual and motor function [42]. They demonstrated immediate improvements in information processing speed in people with multiple sclerosis after a single SVT session lasting between 40 and 50 minutes [42]. Their primary findings were largely consistent with the other studies in healthy adults, which have reported visual improvements following SVT [9,15,16,48,49]. In contrast with the improvements seen in information processing speed, however, no differences were observed in gait and balance outcomes after the single SVT session [42]. However, Symeonido and Ferris found that a single 30-minute session of SVT while walking on a treadmill-mounted balance beam was sufficient to enhance motor performance in young healthy adults [51]. These findings provide strong support for the beneficial training effects of using SVT for task-specific dynamic balance training at least in younger adults and highlight the need for future clinical studies to consider their choice of outcomes in relation to the SVT tasks being provided.

### 4.4 User experience

None of the studies in this review included any participant feedback with regard to the usability of the glasses. A study by Wilkins and colleagues was novel in its inclusion of qualitative data collection methods, but the small sample size (*n =* 3) and lack of detail pertaining to the methodological and analytical processes limits the credibility and transferability of their findings [58]. The lack of work evaluating the acceptability of SVT is perhaps surprising given that wearable comfort is recognised as a key factor affecting end user adoption [59]. Exploring the wearability of the strobe glasses and understanding the needs of the user are particularly important in clinical populations as these are factors likely to influence engagement with SVT within rehabilitation [60–62].

### 4.5 Safety and adverse effects

No adverse events were reported in any of the studies in this review, which is somewhat surprising given that prior research suggests that there may be the potential for individuals to experience irritation or headaches as a result of working under stroboscopic lighting conditions [63,64]. In their methodology, Kim and colleagues did state that “the training session was stopped if dizziness or any adverse event was observed,” but no further reference was made to this in the paper so it is not known how many (if any) participants experienced adverse events as a result of the SVT [50]. Hülsdünker and colleagues reported that “only training drills without head rotation were used” and later recommended that “exercises involving head rotation movements should be avoided because the vestibulo–ocular information mismatch may induce discomfort and increase the risk of injuries” [40]. It is unclear how the authors came to this conclusion, as there was no reporting on adverse events in this paper. Wilkins and Appelbaum and Carrolla and colleagues both acknowledge the potential for “physical hazards” as a result of training in conditions of interrupted vision, but this is again an area that has not been explored in any of the SVT studies to date [1,65].

The potential for experiencing adverse effects such as trips or falls as a result of disrupted vision, or symptoms of discomfort due to the strobe effect, may be even greater in older individuals or those living with long-term conditions because of their dependence on visual input to compensate for sensory or motor losses [66,67]. Future studies therefore need to explore the feasibility of SVT in clinical populations to determine if individuals are able to safely tolerate the strobe effect without experiencing discomfort or unsteadiness.

## 5. Clinical implications

The variation in how SVT research has been carried out means that there is still no consensus with regard to the optimal length of training with strobes (per session and total length) and the most effective strobe frequency at which to train. Furthermore, with all but one study [42] being conducted in healthy cohorts, there is insufficient evidence to inform decisions about the use of SVT in areas of clinical practice involving older adults or individuals with long-term conditions such as Parkinson’s disease, stroke, and MS. Despite these limitations, the findings of this review suggest that SVT may have a role to play in healthcare. SVT can enhance visual skills with some evidence for training effects translating to balance and physical performance in both healthy and clinical populations [42,51]. Although it is still not known whether SVT can have a functional impact, multiple sessions of training (as opposed to a single session) are likely to be required to demonstrate any potential effect on outcomes such as balance and gait in clinical populations [42]. The strobe glasses themselves are lightweight and portable and therefore have the potential to be used, with supervision, in a variety of different settings, which has significant implications for clinical practice as rehabilitation becomes more community based [68]. However, “buy in” to the strobe glasses by health professionals is likely to be affected by the current lack of evidence to support their effectiveness in clinical populations as well as the need to provide one-to-one supervision to individuals while using them [69]. “Buy in” from patient populations may be limited by the fact that the strobe effect could be disorientating for people with existing visual or balance deficits and because of the extra challenge they add to performance.

## Conclusions and recommendations for future research

**Fig 3:**
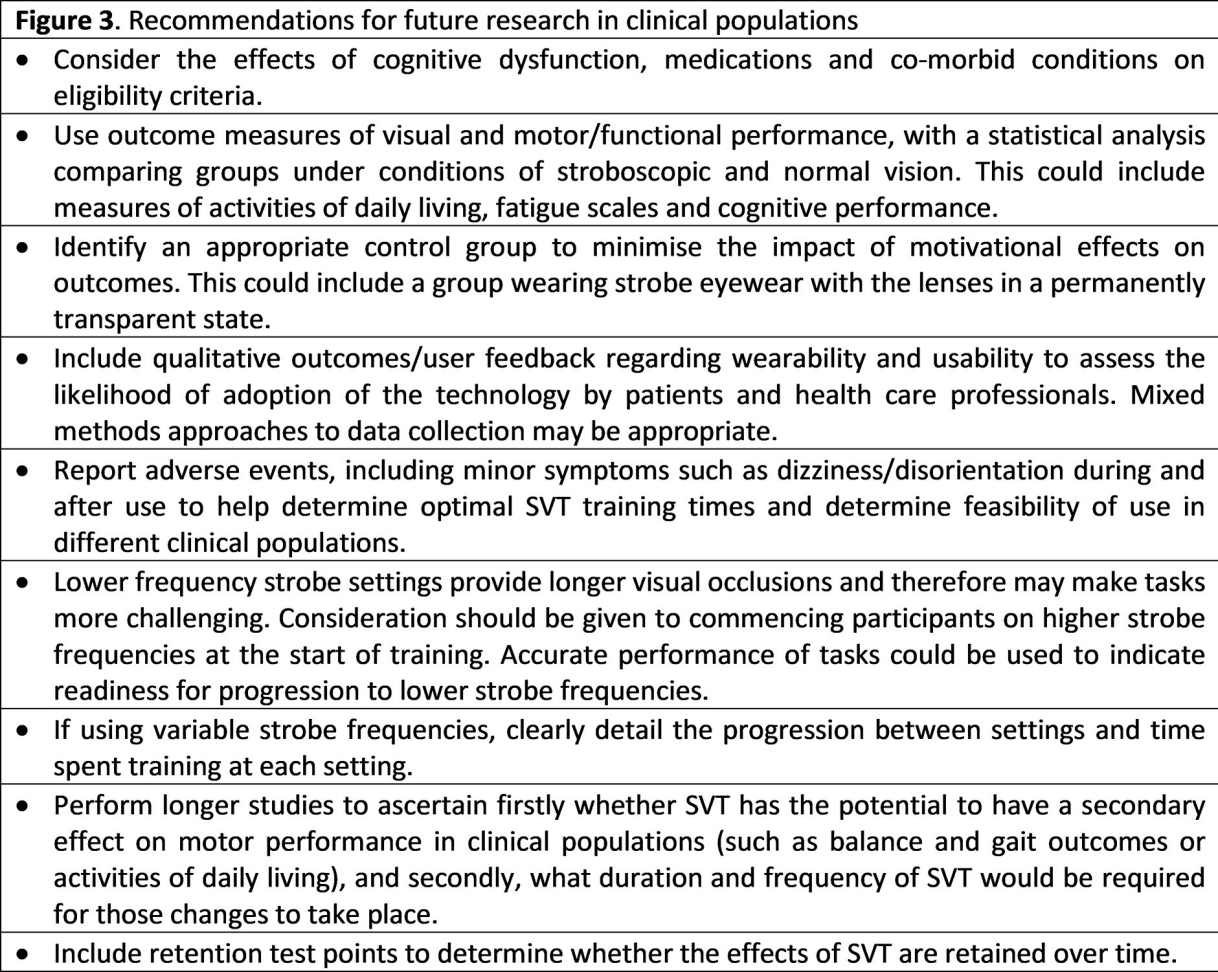
Recommendations for future research in clinical populations.

The purpose of this paper has been to review the scientific literature that has tested the impact of SVT on visual and motor performance in different populations. The functional implications of SVT beyond sport-specific skill performance remain unclear, and the lack of standardised approach to studying the effects of SVT limits understanding and application to clinical practice. Despite these limitations, our review informs this emerging field. Future trials involving SVT should be adequately powered to ensure that statistically significant effects can be detected and preregistered prior to data collection to provide transparency about the research methods used and improve credibility of findings. Fig 3 outlines our recommendations for future research in this field.

## References

[1] Wilkins L, Appelbaum L. An early review of stroboscopic visual training: Insights, challenges and accomplishments to guide future studies. Int Rev Sport Exerc Psychol. 2020;13(1):65–80.

[2] Williams G, Cotter S, Frantz K, Hoffman L, Miller S, Steele G, et al. Vision therapy: Information for health care and other allied professionals–American Academy of Optometry American Optometric Association. Optom Vis Sci. 1999;76(11):739–740. 10566857

[3] Ciuffreda K. The scientific basis for and efficacy of optometric vision therapy in nonstrabismic accommodative and vergence disorders. Optometry. 2002;73(12):735–762. 12498561

[4] Ruiz–Pomeda A, Álvarez–Peregrina C, Povedano–Montero F. Bibliometric study of scientific research on optometric visual therapy. J Opt. 2020;13(3):191–197. doi: 10.1016/j.optom.2019.12.007 32487462 PMC7301205

[5] Schwab S, Memmert D. The Impact of a Sports Vision Training Program in Youth Field Hockey Players. J Sports Sci Med. 2012;11(4):624–631. 24150071 PMC3763307

[6] Zwierko T, Puchalska–Niedbał L, Krzepota J, Markiewicz M, Woźniak J, Lubiński W. The Effects of Sports Vision Training on Binocular Vision Function in Female University Athletes. J Hum Kinet. 2015;49:287–296. doi: 10.1515/hukin-2015-0131 26925183 PMC4723179

[7] Appelbaum L, Erickson G. Sports vision training: A review of the state–of–the–art in digital training techniques. Int Rev Sport Exerc Psychol. 2018;11(1):160–189.

[8] Broadbent D, Causer J, Williams A, Ford P. Perceptual–cognitive skill training and its transfer to expert performance in the field: Future research directions. Eur J Sport Sci. 2015;15(4):322–331. doi: 10.1080/17461391.2014.957727 25252156

[9] Appelbaum L, Schroeder J, Cain M, Mitroff S. Improved Visual Cognition through Stroboscopic Training. Front Psychol. 2011;2:276. doi: 10.3389/fpsyg.2011.00276 22059078 PMC3203550

[10] Elliott D. Intermittent visual pickup and goal directed movement: a review. Hum Mov Sci. 1990;9(3):531–548.

[11] Tsimhoni O, Green P, editors. Visual demand of driving curves determined by visual occlusion. Vision in Vehicles Conference; 1999.

[12] Reschke M, Somers J, Ford G. Stroboscopic vision as a treatment for motion sickness: strobe lighting vs. shutter glasses. Aviat Space Environ Med. 2006;77(1):2–7. 16422446

[13] Reschke M, Krnavek J, Somers J, Ford G, Hwang E, Leigh R, et al., editors. Stroboscopic vision as a treatment for retinal slip induced motion sickness. Proceedings of the First International Symposium on Visually Induced Motion Sickness, Fatigue, and Photosensitive Epileptic Seizures (VIMS2007); 2007.

[14] Holliday J. Effect of stroboscopic vision training on dynamic visual acuity scores: Nike Vapor Strobe Eyewear. 2013.

[15] Appelbaum L, Cain M, Schroeder J, Darling E, Mitroff S. Stroboscopic visual training improves information encoding in short–term memory. Atten Percept Psychophysiol. 2012;74(8):1681–1691. doi: 10.3758/s13414-012-0344-6 22810559

[16] Hülsdünker T, Rentz C, Ruhnow D, Käsbauer H, Strüder H, Mierau A. The effect of 4–week stroboscopic training on visual function and sport–specific visuomotor performance in top–level badminton players. Int J Sports Physiol Perform. 2019;14(3):343–350. doi: 10.1123/ijspp.2018-0302 30160560

[17] Jones C, Carnegie E, Ellison P, editors. The effect of stroboscopic vision training on eye–hand coordination. British Psychological Society (BPS) Division of Sport & Exercise Science Conference; 2016.

[18] Ellison P, Jones C, Sparks S, Murphy P, Page R, Carnegie E, et al. The effect of stroboscopic visual training on eye–hand coordination. Sport Sci Health. 2020;16(3):401–410.

[19] Kim K, Kim J, Oh J, Grooms D. Stroboscopic Vision as a Dynamic Sensory Reweighting Alternative to the Sensory Organization Test. J Sport Rehabil. 2020;1–7. 10.1123/jsr.2019-0466 32473585

[20] Kim K–M, Kim J–S, Grooms D. Stroboscopic vision to induce sensory reweighting during postural control. J Sport Rehabil. 2017;26(5). doi: 10.1123/jsr.2017-0035 28605310

[21] Han S, Lee H, Son S, Hopkins J. The effects of visual feedback disruption on postural control with chronic ankle instability. J Sci Med Sport. 2022;25(1):53–57. doi: 10.1016/j.jsams.2021.07.014 34393051

[22] Suter P, Harvey L. Vision rehabilitation: Multidisciplinary care of the patient following brain injury: CRC Press; 2011.

[23] Wade M, Jones G. The role of vision and spatial orientation in the maintenance of posture. Phys Ther. 1997;77(6):619–628. doi: 10.1093/ptj/77.6.619 9184687

[24] Piñero D. Oculomotor dysfunctions: Evidence–based practice. 2020. 10.1016/j.optom.2020.06.001 PMC7301209 32553271

[25] VanDeMark L. Differences in Postural Control Responses to Levels of Visual Occlusion in Individuals with Chronic Ankle Instability [M.A.]. Ann Arbor: The University of North Carolina at Chapel Hill; 2019.

[26] Beavan A, Hanke L, Spielmann J, Skorski S, Mayer J, Meyer T, et al. The effect of stroboscopic vision on performance in a football specific assessment. Sci Med Footb. 2021;5(4):317–322. doi: 10.1080/24733938.2020.1862420 35077302

[27] Kroll M, Preuss J, Ness B, Dolny M, Louder T. Effect of stroboscopic vision on depth jump performance in female NCAA Division I volleyball athletes. Sports Biomech. 2023;22(8):1016–1026. doi: 10.1080/14763141.2020.1773917 32510290

[28] Grooms D, Chaudhari A, Page S, Nichols–Larsen D, Onate J. Visual–Motor Control of Drop Landing After Anterior Cruciate Ligament Reconstruction. J Athl Train. 2018;53(5):486–496. doi: 10.4085/1062-6050-178-16 29749751 PMC6107770

[29] Dale R, Gollapalli R, Price T, Megahee K, Duncan M, Tolstick N, et al. The effect of visual perturbation upon femoral acceleration during the single and bilateral squat. Phys Ther Sport. 2017;27:24–28. doi: 10.1016/j.ptsp.2017.06.003 28806721

[30] Fransen J, Lovell T, Bennett K, Deprez D, Deconinck F, Lenoir M, et al. The Influence of Restricted Visual Feedback on Dribbling Performance in Youth Soccer Players. Mot Control. 2017;21(2):158–167. doi: 10.1123/mc.2015-0059 27111662

[31] Ballester R, Huertas F, Uji M, Bennett S. Stroboscopic vision and sustained attention during coincidence–anticipation. Sci Rep. 2017;7(1):17898. doi: 10.1038/s41598-017-18092-5 29263340 PMC5738365

[32] Koppelaar H, Moghadam P, Khan K, Kouhkani S, Segers G, van Warmerdam M. Reaction time improvements by neural bistability. Behav Sci. 2019;9(3). doi: 10.3390/bs9030028 30889937 PMC6466602

[33] Liu S, Ferris L, Hilbig S, Asamoa E, LaRue J, Lyon D, et al. Dynamic vision training transfers positively to batting practice performance among collegiate baseball batters. Psychol Sport Exerc. 2020;51:101759.

[34] Appelbaum L, Lu Y, Khanna R, Detwiler K. The Effects of Sports Vision Training on Sensorimotor Abilities in Collegiate Softball Athletes. Athl Train Sports Health Care. 2016;8(4):154–163.

[35] Crémieux J, Mesure S. Differential sensitivity to static visual cues in the control of postural equilibrium in man. Percept Mot Skills. 1994;78(1):67–74. doi: 10.2466/pms.1994.78.1.67 8177690

[36] Boiangin N. Stroboscopic Training Effect on Anticipating the Direction of Tennis Serves [Ph.D.]. Ann Arbor: The Florida State University; 2019.

[37] Edgerton L. The Effect of Stroboscopic Training on the Ability to Catch and Field [M.S.]. Ann Arbor: Arizona State University; 2018.

[38] Chen Y, Chou Y, Hwang I. Reliance on Visual Input for Balance Skill Transfer in Older Adults: EEG Connectome Analysis Using Minimal Spanning Tree. Front Aging Neurosci. 2021;13:632553. doi: 10.3389/fnagi.2021.632553 33613272 PMC7890183

[39] Uzlaşır S, Özdıraz K, Dağ O, Tunay V. The effects of stroboscopic balance training on cortical activities in athletes with chronic ankle instability. Phys Ther Sport. 2021;50:50–58. doi: 10.1016/j.ptsp.2021.03.014 33865218

[40] Hülsdünker T, Gunasekara N, Mierau A. Short–and Long–Term Stroboscopic Training Effects on Visuomotor Performance in Elite Youth Sports. Part 1: Reaction and Behavior. Med Sci Sports Exerc. 2021;53(5):960–972. 33060548 10.1249/MSS.0000000000002541

[41] Hülsdünker T, Gunasekara N, Mierau A. Short–and Long–Term Stroboscopic Training Effects on Visuomotor Performance in Elite Youth Sports. Part 2: Brain–Behavior Mechanisms. Med Sci Sports Exerc. 2021;53(5):973–985. 33060549 10.1249/MSS.0000000000002543

[42] Shalmoni N, Kalron A. The immediate effect of stroboscopic visual training on information–processing time in people with multiple sclerosis: an exploratory study. J Neural Transm. 2020;127(8):1125–1131. doi: 10.1007/s00702-020-02190-2 32279123

[43] Mitroff S, Friesen P, Bennett D, Yoo H, Reichow A. Enhancing ice hockey skills through stroboscopic visual training: a pilot study. Athl Train Sports Health Care. 2013;5(6):261–264.

[44] Wilkins L, Nelson C, Tweddle S. Stroboscopic visual training: A pilot study with three elite youth football goalkeepers. J Cogn Enhanc. 2018;2(1):3–11.

[45] Bennett S, Hayes S, Uji M. Stroboscopic vision when interacting with multiple moving objects: Perturbation is not the same as elimination. Front Psychol. 2018;9:1290. doi: 10.3389/fpsyg.2018.01290 30090080 PMC6068388

[46] Braly A, DeLucia P. Can Stroboscopic Training Improve Judgments of Time–to–Collision? Hum Factors. 2020;62(1):152–165. doi: 10.1177/0018720819841938 31009245

[47] Zavlin D, Chegireddy V, Nguyen–Lee J, Shih L, Nia A, Friedman J, et al. Training Effects of Visual Stroboscopic Impairment on Surgical Performance: A Randomized–Controlled Trial. J Surg Educ. 2019;76(2):560–567. doi: 10.1016/j.jsurg.2018.07.018 30131280

[48] Wilkins L, Gray R. Effects of Stroboscopic Visual Training on Visual Attention, Motion Perception, and Catching Performance. Percept Mot Skills. 2015;121(1):57–79. doi: 10.2466/22.25.PMS.121c11x0 26126135

[49] Smith T, Mitroff S. Stroboscopic training enhances anticipatory timing. Int J Exerc Sci. 2012;5(4):344–353. doi: 10.3758/s13414-012-0344-6 27182391 PMC4738880

[50] Kim K, Estudillo–Martínez M, Castellote–Caballero Y, Estepa–Gallego A, Cruz–Díaz D. Short–Term Effects of Balance Training with Stroboscopic Vision for Patients with Chronic Ankle Instability: A Single–Blinded Randomized Controlled Trial. Int J Environ Res Public Health. 2021;18(10). doi: 10.3390/ijerph18105364 34069907 PMC8157596

[51] Symeonidou E–R, Ferris D. Intermittent Visual Occlusions Increase Balance Training Effectiveness. Front Hum Neurosci. 2022;16:748930. doi: 10.3389/fnhum.2022.748930 35547194 PMC9083907

[52] Senaptec I. Senaptec Products: Senaptec Strobe Classic 2023. Available from: https://senaptec.com/products/senaptec–strobe.

[53] Co Visionup. Ltd. Product Information–Visionup Strobe Glasses 2017. Available from: http://eng.visionup.jp/product–information/.

[54] Hernández–Rodríguez C, Piñero D. Active Vision Therapy for Anisometropic Amblyopia in Children: A Systematic Review. J Ophthalmol. 2020;2020:4282316. doi: 10.1155/2020/4282316 32733699 PMC7376429

[55] Whitecross S. Vision Therapy: Are You Kidding Me? Problems with Current Studies. Am Orthopt J. 2013;63(1):36–40. doi: 10.3368/aoj.63.1.36 24260807

[56] Krzepota J, Zwierko T, Puchalska–Niedbał L, Markiewicz M, Florkiewicz B, Lubiński W. The Efficiency of a Visual Skills Training Program on Visual Search Performance. J Hum Kinet. 2015;46:231–240. doi: 10.1515/hukin-2015-0051 26240666 PMC4519214

[57] Rezaee M, Ghasemi A, Momeni M. Visual and athletic skills training enhance sport p erformance. Eur J Exp Biol. 2012:2.

[58] Johnson J, Adkins D, Chauvin S. A Review of the Quality Indicators of Rigor in Qualitative Research. Am J Pharm Educ. 2020;84(1):7120. doi: 10.5688/ajpe7120 32292186 PMC7055404

[59] Herz M, Rauschnabel P. Understanding the diffusion of virtual reality glasses: The role of media, fashion and technology. Technol Forecast Soc Chang. 2019;138:228–242.

[60] Dunne L, Profita H, Zeagler C. Social aspects of wearability and interaction. Wearable Sensors: Elsevier; 2014. p. 25–43.

[61] Moore K O’Shea E, Kenny L, Barton J, Tedesco S, Sica M, et al. Older Adults’ Experiences With Using Wearable Devices: Qualitative Systematic Review and Meta–synthesis. JMIR Mhealth Uhealth. 2021;9(6):e23832. doi: 10.2196/23832 34081020 PMC8212622

[62] Maranesi E, Di Donna V, Pelliccioni G, Cameriere V, Casoni E, Baldoni R, et al. Acceptability and Preliminary Results of Technology–Assisted Balance Training in Parkinson&rsquo;s Disease. Int J Environ Res Public Health. 2022;19(5). 10.3390/ijerph19052655 PMC8910202 35270348

[63] Shepherd A. Visual Stimuli, Light and Lighting are Common Triggers of Migraine and Headache. J Light Vis Environ. 2010;34(2):94–100.

[64] Inger R, Bennie J, Davies T, Gaston K. Potential Biological and Ecological Effects of Flickering Artificial Light. PLoS ONE. 2014;9(5):e98631. doi: 10.1371/journal.pone.0098631 24874801 PMC4038456

[65] Carrolla W, Fullerb S, Lawrenceb J–M, Osborne S, Stallcup R, Burch R, et al. Stroboscopic Visual Training for Coaching Practitioners: A Comprehensive Literature Review. Int J Kinesiol Sports Sci. 2021;9(4):49–59.

[66] Noell–Waggoner E. Lighting and the Elderly. In: Karlicek R, Sun C–C, Zissis G, Ma R, editors. Handbook of Advanced Lighting Technology. Cham: Springer International Publishing; 2017. p. 847–63.

[67] Lu X, Park N–K, Ahrentzen S. Lighting Effects on Older Adults’ Visual and Nonvisual Performance: A Systematic Review. J Hous Elder. 2019;33(3):298–324.

[68] Chaabene H, Prieske O, Herz M, Moran J, Höhne J, Kliegl R, et al. Home–based exercise programmes improve physical fitness of healthy older adults: A PRISMA–compliant systematic review and meta–analysis with relevance for COVID–19. Ageing Res Rev. 2021;67:101265. doi: 10.1016/j.arr.2021.101265 33571702

[69] AlQudah A, Al–Emran M, Shaalan K. Technology Acceptance in Healthcare: A Systematic Review. Appl Sci. 2021;11(22).

